# The success of various endometrioma treatments in infertility: A systematic review and meta‐analysis of prospective studies

**DOI:** 10.1002/rmb2.12286

**Published:** 2019-06-19

**Authors:** Saeed Alborzi, Ziba Zahiri Sorouri, Elham Askari, Tahereh Poordast, Kefayat Chamanara

**Affiliations:** ^1^ Department of Obstetrics and Gynecology, School of Medicine, Laparoscopy Research Center Shiraz University of Medical Sciences Shiraz Iran; ^2^ Department of Obstetrics & Gynecology, School of Medicine, Reproductive Health Research Center, Alzahra Hospital Guilan University of Medical Sciences Rasht Iran; ^3^ Department of Gynecology and Obstetrics Shiraz University of Medical Sciences Shiraz Iran

**Keywords:** endometriosis, female, infertility, pregnancy rate, reproduction

## Abstract

**Background:**

Endometriosis is seen in 0.5%‐5% of fertile and 25%‐40% of infertile women. To investigate this conflict between gynecologists that ovarian endometriomas should be removed or not before making any decision about pregnancy among infertile women, the authors decided to carry out a systematic review and meta‐analysis to compare the effect of various available therapeutic methods and notice the impact of these options on women's pregnancy rate.

**Methods:**

This review is based on PRISMA recommendations with an electronic search using the following databases: PubMed, Scopus, Google scholar, etc, from 2000 to 2018, in the English language. The studies compare pregnancy rate based on four different treatment types of OMAs between infertile women: (surgery + ART, surgery + spontaneous pregnancy, aspiration ± sclerotherapy + ART, and ART alone).

**Main findings:**

At least eight prospective studies were included, in which 553 infertile women were compared in terms of treatment methods of OMAs before trying to become pregnant.

**Conclusion:**

Treatments are usually based on the patient's clinical condition and must be individual, with the purpose of relieving pain, improving fertility, or both. The authors do not have not any significant difference between our four groups of study; however, the success of surgical procedure compared to other methods was higher and the success of ART alone was the least.

## Abstract 2

Treatments are usually based on the patient's clinical condition and must be individual, with the purpose of relieving pain, improving fertility, or both. We have not any significant difference between our four groups of study; however, the success of surgical procedure compared to other methods was higher and the success of ART alone was the least.

## 1 INTRODUCTION

Endometriosis is seen in 0.5%‐5% of fertile and 25%‐40% of infertile women. Ovarian endometriomas (OMAs) are found in 17%‐44% of women with endometriosis.^1^, ^2^, ^3^, ^4^, ^5^ OMAs diagnosed by ultrasound are identified by the presence of a persistent round shape, thick‐wall cyst (>3 cm), which was filled with a low amount of echogenic fluid.^6^ The presence of OMAs is usually associated with a more advanced stage of disease (stages III and IV of endometriosis according to the American Society for Reproductive Medicine (ASRM) classification), and it predicts a loss of normal pelvic anatomy among these patients.^7^, ^8^, ^9^, ^10^, ^11^, ^12^ Endometriomas can damage ovaries by causing mechanical pulling, regardless of size.^13^, ^14^ Cyst contents include inflammatory factors, proteolytic enzymes, and cellular degrading agents which ultimately cause fibrosis, smooth muscle metaplasia, and decreased cortex‐specific stromal cell.^15^ Moreover, oxidative stress in normal tissue around OMAs has been shown to far more than other benign ovarian cysts.^16^, ^17^, ^18^

The presence of OMAs during assisted reproductive technology (ART) cycles can reduce the actual follicular number by hindering the count and cause difficulty at the time of retrieval.^19^, ^20^, ^21^, ^22^, ^23^ Most infertility specialists refuse to enter and aspirate OMAs during ART procedures for fear of missing an occult early stage of malignancy or of causing a pelvic abscess; however, there are no reports of a missed malignancy to date.^24^

Despite the high prevalence of endometriosis among infertile women and the constant challenge to gynecologists to treat ovarian disease in order to improve fertility, reduce pain symptoms, and prevent the recurrence of disease, an effective treatment for OMAs is still unknown.

Although laparoscopic ovarian cystectomy is still the standard treatment for OMAs and the only way to definitively diagnose it, recent evidence proposes that cystectomy prior to IVF does not improve the clinical fertility rate,^19^, ^20^, ^21^, ^22^, ^23^ and the risk of unwanted and unintentional ovarian tissue removal during cystectomy should not be ignored.^25^

The present study is a systematic review and meta‐analysis which aimed to investigate other methods of therapy on OMAs and compare them in terms of their effects on fertility rate to achieve the best treatment and the best outcome among these patients.

This study compared pregnancy rates based on the following four treatment types among endometriotic infertile women: surgery + ART, surgery + spontaneous pregnancy, aspiration ± sclerotherapy + ART, and ART alone.

## 2 MATERIAL AND METHODS

This study was reported on the basis of the PRISMA checklist.^26^ The population of this review comprised infertile women with ovarian endometrioma. In this systematic review and meta‐analysis, the success rates of various treatments of endometriomas for fertility rate and clinical pregnancy rate were determined.

An electronic search was conducted on the PubMed, Scopus, Google Scholar, EMBASE, and the Cochrane Library databases for articles published from 2000 to 2018, using a combination of controlled vocabulary and free text in the English language with the following keywords: surgical and nonsurgical treatment of endometrioma, infertility and pregnancy rate, and assisted reproduction therapy. A manual search of all references was also performed.

All prospective studies reporting samples with an age range of 15‐45 years, fertility rate, treatment description, and clinical pregnancy number (from when the embryo's heartbeat appeared in the ultrasound) were included; other reviews, case studies, retrospective studies, studies that did not explain the method of treatment, and those including patients with previous endometriotic surgery were excluded. Studies of women who had received medical hormonal therapy prior to treatment and those that did not report sample size, power description, or outcome were also excluded.

All articles were independently evaluated by two reviewers based on the inclusion and exclusion criteria. Both reviewers summarized all data extracted from the articles, and where the data were inconsistent, problems were resolved by arbitration and the comments of a third reviewer. To assess the methodological quality of every article that was included in this research, the US National Institute of Health, National Heart, Lung, and Blood Institute quality assessment tool for observational cohort and cross‐sectional studies was used.^27^ This tool measures 14 different criteria which are used to give each study an overall quality rating of good, fair, or poor. All articles included in this research had good quality. The current results according to the mentioned checklist are summarized in Table 1.

**Table 1:** Quality of studies using NIH's quality assessment for cohort and cross‐sectional studies

Criteria	Bila et al	Alborzi et al	Busacca et al	Alborzi et al	Pabuccu et al	Demirol et al	Fisch et al	Suganuma et al
1. Was the research question or objective in this article clearly stated?	✓	✓	✓	✓	✓	✓	✓	✓
2. Was the study population clearly specified and defined?	✓	✓	✓	✓	✓	✓	✓	✓
3. Was the participation rate of eligible persons at least 50%?	✓	✓	✓	✓	✓	✓	✓	✓
4. Were all the patients selected or recruited from the same or similar populations (including the same time period)? Were inclusion and exclusion criteria for being in the study prespecified and applied uniformly to all participants?	✓	✓	✓	✓	✓	✓	✓	NR
5. Was a sample size justification, power description, or variance and effect estimates provided?	NR	✓	NR	NR	NR	✓	✓	✓
6. For the analyses in this article, were the exposure(s) of interest measured prior to the outcome(s) being measured?	✓	NR	NR	NR	NR	NR	NR	NR
7. Was the time frame sufficient so that one could reasonably expect to see an association between exposure and outcome if it existed?	✓	**×**	✓	✓	✓	✓	✓	✓
8. For exposures that can vary in amount or level, did the study examine different levels of the exposure as related to the outcome (eg, categories of exposure, or exposure measured as continuous variable)?	✓	✓	✓	✓	✓	✓	✓	✓
9. Were the exposure measures (independent variables) clearly defined, valid, reliable, and implemented consistently	✓	✓	✓	✓	✓	✓	✓	✓
10. Was the exposure(s) assessed more than once over time?	✓	NR	NR	NR	NR	NR	NR	NR
11. Were the outcome measures (dependent variables) clearly defined, valid, reliable, and implemented consistently across all study participants?	✓	✓	✓	✓	✓	✓	✓	✓
12. Were the outcome assessors blinded to the exposure status of participants?	NR	NR	NR	NR	NR	NR	NR	NR
13. Was loss to follow‐up after baseline 20% or less?	✓	✓	✓	✓	✓	✓	✓	✓
14. Were key potential confounding variables measured and adjusted statistically for their impact on the relationship between exposure(s) and outcome(s)?	✓	✓	✓	✓	✓	×	×	×

Abbreviation: NR, not reported.

To analyze the clinical pregnancy rate, we extracted the data on total number of women undergoing all ART methods and a group of women who got pregnant spontaneously after an operation for OMAs.

The results were reported with 95% confidence interval (CI).^28^ Cochran's *Q* test and the *I* ^2^ index were used to report heterogeneity. An *I* ^2^ index value of 0%‐50% indicated low heterogeneity, and a value >50% demonstrated high heterogeneity.^29^, ^30^ If *I* ^2^ > 50%, the random effect was used to interpret the results.

Because the number of studies was less than 10, the publication bias was not calculated. The data were analyzed using STATA (12.2 version) and MedCalc (18.9.1 version) software.

## 3 RESULTS

**Figure 1:**
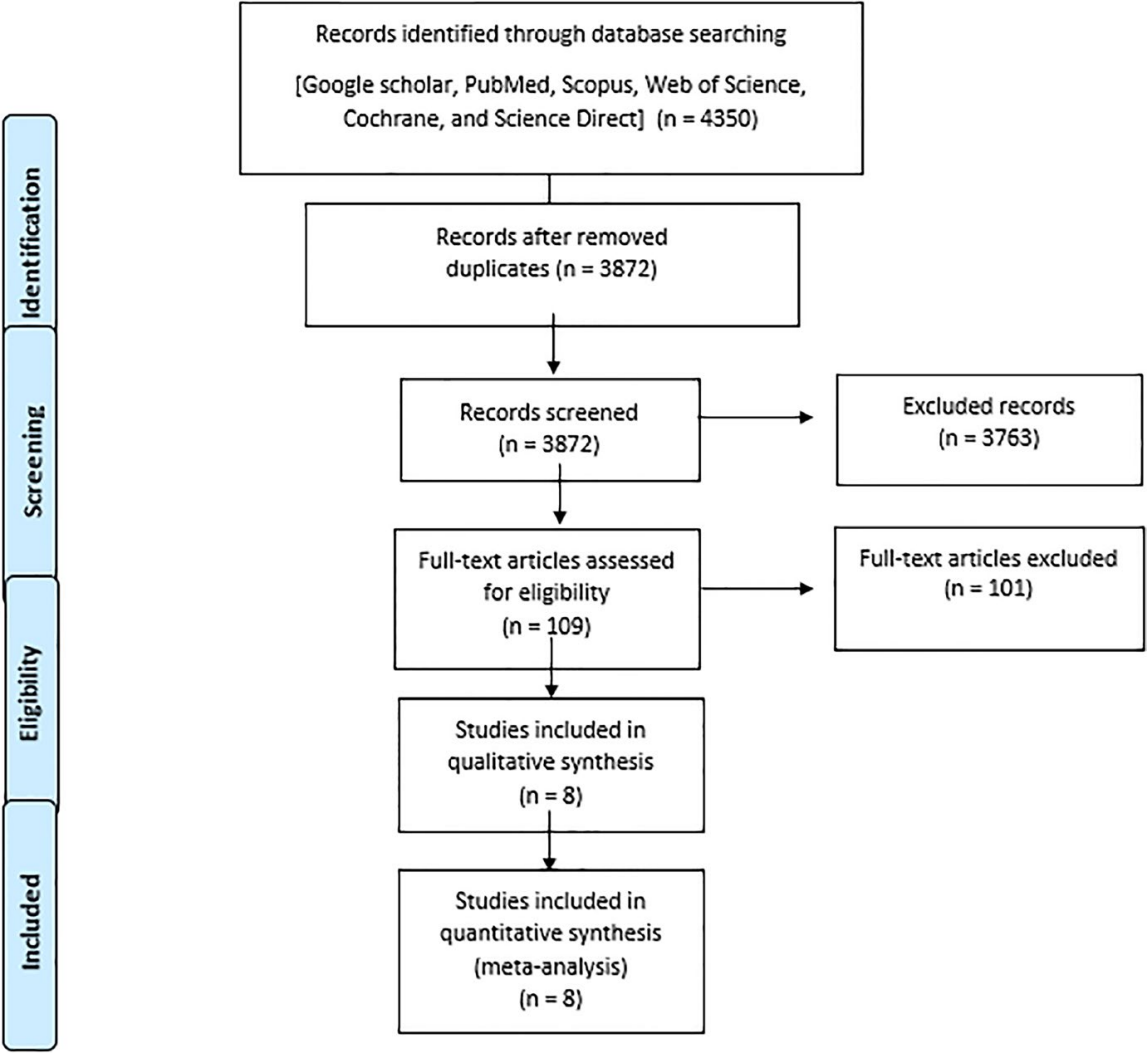
Flowchart describing the study design process

In the first phase of the search process, 4350 articles were identified. After a review of the articles, 1190 inappropriate or repetitive articles were excluded. Finally, after reviewing the content and quality of the remaining articles, 8 were found to be eligible and chosen for this study (Figure 1).

All data about the authors, places and period of research, studies and diagnostic methods, treatment methods, and outcomes of research are given in Table 2.^6^, ^22^, ^31^, ^32^, ^33^, ^34^, ^35^, ^36^

**Table 2:** Main characteristics of the included studies on treatment methods of endometriosis and its effect on clinical pregnancy rate as an outcome

Article's	Population	Endometriosis groups	Diagnosis	Design	Outcomes	References
Bila et al 2018, Serbia N = 77	73 patients with primary infertility caused by endometriosis were subjected to 77 IVF/ICSI cycles at tertiary institutions for a period of 5 y in Medical faculty University of Belgrade, Serbia.	Patients were classified into two groups. (Group I, n = 46) Some kind of endometriosis treatment had previously been applied before the introduction to the IVF/ICSI, with two subgroups: (A) combination of surgical and medical treatment (n = 25) and (B) only surgically treated (n = 21) and (Group II, n = 27) patients were immediately subjected to the IVF/ICSI procedures.	Ultrasound and diagnostic laparoscopy without intervention	Prospective cohort study	Pregnancy rate	33
Alborzi et al, 2007, Iran N = 81	To compare the ovary that had been operated on (cystectomy or fenestration coagulation) with the unoperated contralateral one of the same patients with respect to COH. From January 2002 to September 2005, 81 patients with infertility due to endometriosis were subjected for study.	Patients were divided into three groups: Group 1 (n = 24) and Group 2 (n = 41) with unilateral OMAs underwent fenestration and coagulation or the cystectomy. Group 3 (n = 16) patients with bilateral OMAs that ovarian cystectomy was done in one side and fenestration and coagulation in other side. (We omitted this group from our review). All patients referred for COH after surgery.	Ultrasound	Prospective randomized design study	Ovarian response to COH after surgery Pregnancy rate	34
Busacca et al, 2006, Italy N = 126	Patients who had been operated on for bilateral ovarian endometriosis between January 1995 and December 2003 were included.	Sixty‐five women of these population tried to become pregnant after surgery, 43 of them had at least 1 pregnancy (66.2%). Between 18 infertile women at the time of surgery, 9 of them subsequently conceived (50%).	Ultrasound and histopathology confirmation	Prospective study	Postsurgical ovarian failure Pregnancy rate	35
Alborzi et al, 2004, Iran N = 100	To determine the difference between two laparoscopic methods for the management of endometriomas with regard to the recurrence of signs and symptoms and pregnancy rate in 100 patients with unilateral OMAs from March 1998 to December 2001. Patients followed up at 3, 6, 9, 12, 18, and 24 months after surgery.	Patients divided into two groups: Group 1 (n = 52) in the cystectomy group and Group 2 (n = 48) in fenestration and coagulation group. There were 19 pregnancies among 32 infertile patients in Group 1 (59.4%) and 7 pregnancies among 30 infertile patients in Group 2 (23.3%) after 1‐y follow‐up without any medications.	Ultrasound and histopathology confirmation	Prospective, randomized clinical trial	Recurrence of signs and symptoms and pregnancy rate according to two laparoscopic methods.	6
Pubuccu et al, 2004, Turkey N = 171	Between January 1999 and August 2002, 171 patients went through 171 ICSI cycles with ejaculated sperm at the Assisted Reproduction Unit of the Gulhane School of Medicine Department of Obstetrics and Gynecology	Patients were divided into four groups: aspiration of OMAs at the beginning of controlled ovarian hyperstimulation (COH) in patients with ovarian OMAs and no history of previous surgery (n _ 41) (Group 1); nonaspirated OMAs (n _ 40) (group 2); history of ovarian surgery for OMAs in patients without ovarian OMAs at the beginning of COH (n _ 44) (Group 3); and tubal factor infertility (n _ 46) (control Group 4). In Group 3, we have 23 bilateral and 21 patients with unilateral OMA, and endometriosis was resected twice in 11 women and once in 33 women.	Ultrasound and histopathology confirmation	Prospective study.	Intracytoplasmic sperm injection (ICSI) outcomes Pregnancy rate	31
Demirole et al, 2006, Turkey N = 99	Ninety‐nine patients with unilateral OMAs with the diameter between 3 and 6 cm were referred to an intracytoplasmic sperm injection (ICSI) cycle at the Clinic Women Health, Infertility and IVF Centre, Ankara, Turkey, between January 2001 and March 2005	Patients were divided into two groups: Patients in Group I (49 patients) underwent conservative ovarian surgery before the ICSI cycle and patients in Group II (50 patients) underwent the ICSI cycle directly. The rate of male factor infertility was similar in both groups (59.18 vs 62.00). All patients were stimulated with luteal long protocol.	Ultrasound	Prospective, randomized study	Intracytoplasmic sperm injection (ICSI) outcomes ‐Pregnancy rate	32
Fisch et al, 2004, US N = 32	Infertile women (n = 32) with OMAs were offered sclerotherapy in lieu of surgery. Patients’ cysts ranged in size between 1.5 and 6.0 cm. Patients were eligible for IV treatment if OMAs were resolved 6 wk after sclerotherapy.	In 24 (75%) of 32 patients, the OMAs were completely resolved at the 6‐wk follow‐up. Eight patients had a residual simple cyst that required repeat aspiration before complete resolution. Two patients needed an additional treatment with TCN. We have 57% (n = 16) ongoing pregnancy rate after sclerotherapy between 28 infertile women.	Ultrasound	Prospective, cohort.	Resolution of OMA Subsequent IVF pregnancy rate	22
Suganum et al, 2002, Japan N = 79	79 infertile women with OMAs who underwent IVF‐ET were selected to evaluate the effect of pretreatments for OMAs before IVF cycle in Japan.	79 infertile women with OMAs who underwent IVF‐ET were divided into 3 groups as follows: (1) 62 IVF cycles in 36 patients who underwent laparotomy or laparoscopy (“Surgery” group), (2) 35 cycles in 23 patients for whom the OMA content was aspirated and treated with or without alcohol fixation (“Cyst aspiration” group), and (3) 30 cycles in 20 patients who did not undergo pretreatment and confirmed ovarian endometriosis at oocyte retrieval (“No treatment” group). Ovarian hyperstimulation was performed following a short, long, and ultralong protocols.	Ultrasound	Prospective study	IVF‐ET outcome Pregnancy rate	36

### 3.1 Analytical results

This study included a survey of clinical pregnancy rates among 553 infertile women with endometriosis which were classified into four groups based on their treatment type: Group 1 (243 patients, 43.9%) = surgery + ART; Group 2 (80 patients, 14.4%) = surgery + spontaneous pregnancy; Group 3 (142 patients, 25.6%) aspiration ± sclerotherapy + ART; and Group 4 (88 patients, 15.9%) ART alone **(**Table 3).

**Table 3:** Description of patients in relation to pregnancy outcome and endometriosis therapy

Methods of intervention	Number of infertile women	Number of pregnancy	Pregnancy rate %	Duration of follow‐up
Surgery + ART	243	93	38.7	1‐2 cycles
Surgery	80	35	43.7	2 y
Aspiration ± sclerotherapy + ART	142	56	39.4	1‐2 cycles
ART	88	26	29.5	1‐2 cycles
Total	553	210	37.9	‐

The surgery group included those who underwent a procedure for cystectomy (269 patients) and those who underwent fenestration and coagulation of cyst (64 patients). Assisted reproductive technology (ART) included three methods: in vitro fertilization/intracytoplasmic sperm injection (IVF/ICSI) (426 patients, 86.7%) and intrauterine insemination (IUI) (65 patients, 13.2%).

The IUI procedure was performed only in a subgroup of patients undergoing surgery (Group 1). In other groups, the term ART was used to refer to IVF/ICSI methods. All patients who were nominated for assisted reproductive technology received just 1 or 2 cycles of embryo transfer or at least 2 cycles of IUI after controlled ovarian stimulation (COH) with human menopausal gonadotropin (HMG) ampules.

For patients who were monitored for spontaneous pregnancy, a period of 12 months was given to get pregnant (80 patients, 14.4%).

The surgical technique for cystectomy involved stripping the cyst wall from the ovarian parenchymal through traction and countertraction in opposite directions by laparoscopic method. Except for the study of Suganum et al,^36^ who included ovarian endometrioma surgery using both laparoscopy and laparotomy methods on 32 patients, gentle bipolar coagulation was performed to the ovarian struma when necessary, and the inner linings of the cyst wall were sent for histopathologic examination.

In the fenestration and coagulation technique, a 1.5 × 1.5‐centimeter biopsy from the inner lining of the cyst was taken, and then coagulation of the inner cyst wall was performed with bipolar electrocautery.

In the aspiration ± sclerotherapy + ART group, all endometriomas within the size range of 1.5 and 6.0 cm were aspirated and flushed with sterile saline until the aspirated fluid became clear under the guidance of vaginal ultrasound. The cyst contents were sent for pathologic review. After that, sometimes 96% alcohol or other materials used for sclerotherapy were instilled into the cyst and led to the destruction of the cyst wall. The cyst could be aspirated at the start of the IVF cycle^31^ or at the time of ovum retrieval.^32^ If alcohol or other materials are injected into the cyst, the patient should wait at least 4 to 6 weeks before starting IVF cycles to ensure the effectiveness of the treatment, and the procedure should be occasionally repeated.

There was no significant difference between the four groups in terms of age (mean = 31.1 years) or BMI (<30). All patients had a regular menstruation period, and the OMAs were approximately the same size (mean = 38.4 mm) (*P* < 0.0001).

In terms of severity of endometriosis, 333 patients were in stage III or stage IV based on the ASRM classification that was mentioned in only four articles.^6^, ^33^, ^34^, ^35^

### 3.2 Clinical pregnancy rate

The clinical pregnancy rate, an outcome based on the four groups, was extracted from the 8 eligible studies. The cumulative pregnancy in the surgery group (Group 2) and clinical pregnancy per cycle in other groups were measured and compared. The results showed no significant differences among the four groups in clinical pregnancy rates. The results are as follows: Group 1 (surgery + ART): pregnancy rate = 38.3% (CI: 32.3‐44.7); Group 2 (surgery alone): pregnancy rate = 43.8% (CI: 22.5‐66.4); Group 3 (aspiration ± sclerotherapy + ART): pregnancy rate = 40.8% (CI: 27.7‐54.6); and Group 4 (ART alone): pregnancy rate = 32% (CI: 15.0‐52.0).

Comparing these groups, it seems ART alone in infertile endometriotic women is associated with fewer pregnancies than other therapeutic methods (Table 4).

**Table 4:** Med Calc of the systematic review and meta‐analysis based on methods and pregnancy outcome

	Sample size	Proportion (%) Pregnancy	95% CI	Weight (%)		*I* ^2^	Sig. diff
				Fixed	Random		
Study (Group 1 = surgery + ART)							
Pabuccu et al	44	27.273	14.958‐42.790	18.07	17.74		
Demirol et al	49	34.694	21.672‐49.639	20.08	18.93		
Suganuma et al	36	50.000	32.922‐67.078	14.86	15.62		
Bila et al	49	48.980	34.425‐63.662	20.08	18.93		
Alborzi et al	41	36.585	22.123‐53.064	16.87	16.98		
Alborzi et al	24	29.167	12.615‐51.095	10.04	11.81		
Total (fixed effects)	243	38.380	32.310‐44.731	100.00	100.00	35.16	0.1729
Study (Group 2 = surgery)							
Alborzi et al	32	59.375	40.645‐76.302	39.76	34.93		
Alborzi et al	30	23.333	9.934‐42.284	37.35	34.50		
Busacca et al	18	50.000	26.019‐73.981	22.89	30.58		
Total (random effects)	80	43.848	22.504‐66.466	100.00	100.00	77.36	0.0121
Study (Group 3 = aspiration±sclerotherapy + ART)							
Pabuccu et al	41	24.390	12.363‐40.305	28.77	26.73		
Fisch et al	28	57.143	37.179‐75.538	19.86	23.38		
Suganuma et al	23	47.826	26.820‐69.412	16.44	21.55		
Demirole et al	50	38.000	24.650‐52.825	34.93	28.34		
Total (random effects)	142	40.853	27.791‐54.609	100.00	100.00	64.18	0.0389
Study (Group 4 = ART)							
Bila et al	28	25.000	10.691‐44.872	31.87	33.42		
Pabuccu et al	40	20.000	9.052‐35.648	45.05	36.26		
Suganuma et al	20	55.000	31.528‐76.942	23.08	30.33		
Total (random effects)	88	32.088	15.078‐52.024	100.00	100.00	73.40	0.0233

### 3.3 Fertilization rate and other outcomes

According to the findings summarized in Table 5, three studies examined the fertilization rate of surgery + ART, aspiration + ART, and ART alone. The results did not show any significant differences among the three groups, but surgery and aspiration of the cyst before starting ART procedures seemed to increase the fertilization rate without any difference in duration of stimulation of ovaries or in required dosage of hormonal drugs during the ART procedure. Only one study was found regarding postsurgical treatment with GNRH‐agonist drugs, especially in moderate‐to‐severe endometriotic and symptomatic women, and their effects on pregnancy rate.^33^

**Table 5:** Description of the cycle (IVF/ICSI) in relation to outcome

Author name	Treatment methods	Basal FSH	Basal E2	Dose of gonadotropins (IU)	Duration of stimulation	Estradiol at the day of HCG	Total oocyte number	MII oocyte number	Implantation rate	Fertilization rate (%)	Clinical pregnancy rate
Bila et al	Surgery + ART	7.43 ± 3.78	41.75 ± 21.13	‐	‐	‐	‐	‐	‐	‐	24 (77.4)
	ART	6.81 ± 3.24	49.42 ± 41.39	‐	‐						7 (22.6)
Pabuccu et al	Aspiration + ART	7.2 ± 1.7	62.4 ± 17.5	2512.5 ± 547.5	10.9 ± 1.4	1632 ± 670	‐	6.1 ± 1.1	13	72 ± 10	10 (24)
	ART	6.8 ± 1.8	66.7 ± 18. 4	2760 ± 742.5	11.6 ± 1.6	946.7 ± 264	‐	5.6 ± 1.2	12	68 ± 16	8 (20)
	Surgery + ART	7.1 ± 1.7	62.4 ± 16.6	2490 ± 622.5	10.5 ± 1.6	1196 ± 444	‐	5.7 ± 1.13	18	72 ± 13	12 (25)
Demirol et al	Surgery + ART	8.2 ± 0.36	‐	4575 ± 530	14 ± 2.5	1170 ± 417	7.8 ± 3	‐	16.5	86.2	17 (34.4)
	Aspiration + ART	7.9 ± 0.36	‐	3675 ± 792.58	10.8 ± 2.6	1680 ± 428.69	8.6 ± 2.82	‐	18.5	88.3	19 (38.2)
Suganuma et al	ART	‐	‐	‐	‐	‐	9.7 ± 6.7	8 ± 5. 4	‐	56.5	11 (55)
	Surgery + ART	‐	‐	‐	‐	‐	7.2 ± 6.2	5.7 ± 4.8	‐	56.8	18 (50)
	Sclerotherapy + ART	‐	‐	‐	‐	‐	6.6 ± 5.5	5.1 ± 3.7	‐	67.4	11 (47.8)

Only three articles mentioned the duration of infertility in the absence of associated infertility factors; Billa et al,^33^ and Alborzi et al^6^ mentioned a duration of more than one year, and Pubuccu et al^31^ reported a mean period of infertility of 5 years. Moreover, they had better outcomes of pregnancy in the group with a shorter infertility duration.

In most groups, unilateral rather than bilateral OMAs were observed (n = 406 and n = 82,), respectively.

## 4 DISCUSSION

This systematic review and meta‐analysis aimed to determine the success rate of endometrioma treatment in infertility based on prospective studies published between 2000 and 2018. The results showed that the most commonly used methods were surgery + ART (43.94%), aspiration ± sclerotherapy + ART (25.67%), ART alone (15.91%), and surgery (14.46%) (Table 3).

Despite the high prevalence of endometrioma and gynecologists’ continual encounters with patients with this problem, there is still no definitive treatment that results in complete recovery and discontinuation of symptoms.

Some OMAs do not have any specific symptoms. Currently, they are treated individually and based on the patient's condition. In recent decades, surgery and laparoscopic excision have been used to treat OMAs; however, recent observations have shown that surgical excision of the OMAs can reduce ovarian reserve and subsequently affect the pregnancy process. In a review study by Streuli et al,^37^ surgical excision of OMAs was reported to be capable of negatively affecting fertility. In their report, Bussaca et al^35^ also referred to three cases (2.4%) of premature ovarian failure (POF) that presented immediately after ovarian surgery for endometriosis (all cases in this article had bilateral OMAs) in patients aged 31, 33, and 39 years who had normal, regular menstruation before their operations.

This issue has also been addressed in the International Standard Guides. The 2013 guideline of ESHRE states that cysts 3 cm or larger must be surgically excised when endometrioma is detected so as not to miss a malignancy in rare cases.^25^ However, there are still many doubts about this decision. Of course, the very low malignancy rates in typical OMAs and the reduced fertility rates caused by the operation must be compared, and before deciding on any surgical intervention, a solution to promote fertility must be sought.

Studies have shown that the rate of endometrioma recurrence after laparoscopic ovarian cystectomy is 6%‐67%, while the rate of recurrence after aspiration is 28%‐98%.^25^, ^38^, ^39^, ^40^ In another study, Noma and Yoshida demonstrated that the rates of recurrence after surgery and sclerotherapy were 3.8% and 14.9%, respectively.^41^ Because of the short duration of the included studies, only one detailed account of the possibility of a recurrence of cysts following various surgical procedures was found. Alborzi et al^6^ compared the recurrence of symptoms (pain, dysmenorrhea) and the reoperation rate during the follow‐up period between two different surgical procedures for OMAs. The results of their study showed significantly lower recurrence and reoperation rates (9.5% vs. 15.8%) in the cystectomy group compared with the fenestration and coagulation group (3.5% vs. 11.4%) after 2 years.^6^ Busacca et al^35^ reported 4.8% recurrence of OMAs after cystectomy during 4 years of follow‐up.

Since endometrioma is a pseudocyst, the risk of removing normal tissue during surgery is high. Therefore, there are concerns about reduced fertility and IVF outcome. Hence, a number of studies have examined anti‐Mullerian hormone markers (AMH) and antral follicle count (AFC) to predict ovarian reserve. AFC is also thought to be reduced in these patients due to inflammation caused by endometrioma.^41^ In a systematic review and meta‐analysis of 14 papers (597 patients) conducted in 2014, the AFC did not change significantly after surgery in endometriotic women.^42^, ^43^ Probably due to the presence of endometrioma, AFC had been underestimated prior to surgery. In a systematic review and meta‐analysis of eight prospective cohorts (237 patients) by Raffi et al,^44^ however, it was found that serum AMH levels decreased significantly after surgery—1.13 ng/mL (95% CI, −0.37 to −1.88), despite the high heterogeneity reported in this study. Similarly, Alborzi et al^20^ reported a decrease in AMH level and an increase in FSH after ovarian endometrioma surgery in 215 women.

In the current systematic review and meta‐analysis, surgery following aspiration ± sclerotherapy + ART was the most successful treatment based on clinical pregnancy outcome. In a systematic review and meta‐analysis in 2017, Cohen et al showed that endometrioma recurrence was reduced after several sclerotherapy sessions with ethanol. The pain was also alleviated by 68%‐96%, and the pregnancy rate in this group was similar to that in the surgery group. Cohen et al^45^ included retrospective studies in their meta‐analysis as well, and in their analysis, the risk of OMAs recurrence was significantly higher in women treated with ethanol washing than in the group with ethanol retention. As in the current study, Cohen et al reported similar pregnancy rates in the cystectomy and sclerotherapy groups.

In the present systematic review and meta‐analysis, the pregnancy rate of ART after surgery was 38.3%, which was lower after ART alone than other methods. In the study of Alkudmani et al, the time for IVF after surgery was known to be effective. After controlling the age and the stage of the endometriosis, the authors showed that the highest pregnancy rate was in patients who started IVF 6 to 25 months after surgery rather than the proportion of patients over 25 months after surgery.^46^ In addition, Billa et al 2018 reported a higher pregnancy rate in the surgery + ART group who received 3‐6 months of repressive therapies according to the stage of endometriosis rather than the surgery group who did not receive repressive therapy before ART (44% vs. 21%). They also had better results than in the ART alone group.^33^ This point may have not been considered in the studies evaluated in this meta‐analysis. In another study on 61 women, Geber et al showed that surgery + ART may not be suitable for women over 35 years of age and may cause more complications; however, a lower pregnancy rate was observed in patients who had undergone previous ovarian surgery. This difference was not statistically significant compared with the control group. The authors recommended that for infertile patients, especially those over 35 years of age, nonsurgical treatment might be a better option to avoid a reduction in the ovarian response.^47^ Physicians must consider all of these factors for postsurgical IVF, keeping in mind each patient's specific condition.

The lowest pregnancy rate was observed for ART group (32%), which could be explained by the presence of endometrioma, because it reduces the number of oocytes and makes them less accessible. Moreover, it makes oocyte pickup more difficult, which causes an increase in the cancelation rate and reduces embryo transfer.

In the current systematic review and meta‐analysis of surgical procedures, cystectomy was found to be more successful than fenestration and coagulation, in terms of pregnancy rate and recurrence of the cysts, if the significant difference in the sample size of the two groups is excluded.

Hart et al^48^ conducted a systematic review and meta‐analysis of two randomized control trials (RCTs) and 164 women and showed that cystectomy was associated with lower rates of dysmenorrhea, dyspareunia, and non‐menstrual pelvic pain risk compared with fenestration and coagulation.

In another systematic review and meta‐analysis, Dan and Limin^49^ found that the odds ratio of pain and dysmenorrhea for the cystectomy was far less than for fenestration and coagulation. In the study of Alborzi et al,^6^ cystectomy was shown to be preferable to fenestration and coagulation because of the reduction in recurrence and symptoms, need for subsequent surgeries, and the increased cumulative pregnancy rate (43% vs 13.7%), but in terms of ovarian response, both groups had a similar response. Another reason is that in the fenestration and coagulation method, ovarian cautery can cause normal tissue loss around the coagulated cyst and eventually damage the ovary, which may reduce the pregnancy rate compared with cystectomy.

In all studies for IVF/ICSI, the use of long protocol had a very clear and more significant effect on total retrieved oocytes and pregnancy rate than other protocols.

## 5 CONCLUSION

Although treating endometrioma is a permanent problem for gynecologists, the choice of the best treatment remains a challenge for them. Treatments are usually based on the patient's clinical condition and must be individual, with the purpose of relieving pain, improving fertility, or both. No significant difference was observed among the four groups in the current study; however, the success rate of the surgical procedure compared with the other methods was higher; the success rate of ART alone was the lowest.

The severity of the illness and the patient's condition were not absolutely clear in the studies, which made it difficult to make definitive conclusions. Also in the surgery treatment, the cumulative pregnancy rate was reported, while in the ART method, pregnancy was reported per cycle for each patient.

## 6 LIMITATIONS

As with other meta‐analyses, this study had some limitations. One reason for disagreement over the success of treatment is the high heterogeneity of these papers. In the current study, there is a high level of heterogeneity in the design of the studies and the measurement of the index.

The small sample sizes, inadequate or inappropriate follow‐ups, and unclear inclusion and exclusion criteria in the studies also limited the present study and could partially affect the results. The impossibility of conducting interventional studies is one of the main problems in the treatment of endometrioma.

## DISCLOSURES

*Conflict of interest*: Saeed Alborzi, Ziba Zahiri Sorouri, Elham Askari, Tahereh Poordast, and Kefayat Chamanara declare that they have no conflict of interest. *Human/animal rights statements and informed consent*: This article does not contain any studies with human and animal patients performed by any of the authors.

## ETHICAL APPROVAL

Because this study only reviews and compares the previously published articles and does not contain any studies with human patients, the approval by Ethics Committee is not applicable.
